# Similar overall survival with reduced vs. standard dose bevacizumab monotherapy in progressive glioblastoma

**DOI:** 10.1002/cam4.2616

**Published:** 2019-11-22

**Authors:** Jack Patrick Gleeson, Fergus Keane, Niamh M. Keegan, Emin Mammadov, Emily Harrold, Abdullah Alhusaini, Jeffrey Harte, Austin Eakin‐Love, Philip J. O'Halloran, Stephen MacNally, Bryan T. Hennessy, Oscar S. Breathnach, Liam Grogan, Patrick G. Morris

**Affiliations:** ^1^ Medical Oncology Department Beaumont Hospital Dublin Ireland; ^2^ Medical Oncology Department Memorial Sloan Kettering Cancer Center New York NY USA; ^3^ Medical Oncology Department Mater Misericordiae University Hospital Dublin Ireland; ^4^ Royal College of Surgeons of Ireland (RCSI) Dublin Ireland; ^5^ Neurosurgical Department Beaumont Hospital Dublin Ireland; ^6^ Cancer Clinical Trials and Research Unit RCSI Hospital Group Beaumont Hospital Dublin Ireland

**Keywords:** bevacizumab, cost analysis, glioblastoma, MGMT, overall survival, reduced dose

## Abstract

**Introduction:**

Bevacizumab has demonstrated activity in glioblastoma (GBM), but the true benefits and optimal dose‐schedule are debated. A lower dose‐schedule than standard‐dose bevacizumab (10 mg/kg 2‐weekly) might offer similar benefits with lower costs. At our Institution, patients are randomly assigned at time of primary diagnosis to Neuro‐Oncologists, who have varying practices in terms of bevacizumab dose‐schedule upon progression.

**Methods:**

In a retrospective analysis we examined overall survival (OS), measured from first administered bevacizumab dose until death, according to dose‐schedule. Patients with de novo WHO Grade IV GBM who received standard‐ or reduced‐dose (5 mg/kg 2‐weekly) bevacizumab were included. MGMT methylation status and time from diagnosis to bevacizumab start were examined as prognostic variables. Clinical benefit and a comparative cost analysis were assessed.

**Results:**

In total, 1127 bevacizumab doses were administered to 118 patients [Median: 7, Range: 1‐44]. Median OS (mOS) was 5.8 months. 69 (59%) patients received standard‐dose bevacizumab (mOS: 5.97 months) and 49 patients received reduced‐dose (mOS: 5.7 months). No statistically significant difference in OS between dosing schedule was seen (HR: 1.11, *P*‐value: .584). Patients with MGMT methylated tumors (43%) had improved OS compared to those with unmethylated tumors; 7.03 vs 4.97 months (HR: 0.61, *P*‐value: .027). If all patients were treated with reduced‐dose bevacizumab, an estimated €2.4M cost reduction would be observed.

**Conclusions:**

In this retrospective study, reduced‐dose bevacizumab schedule resulted in similar OS to standard‐dose bevacizumab monotherapy with substantial cost savings. MGMT methylation appears to convey a survival benefit in the setting of bevacizumab treatment for progressive GBM.

## Graphical abstract

In this analysis, 118 de novo WHO grade IV glioblastoma patients were analysed to assess the impact of standard vs reduced dose bevacizumab on overall survival and a cost analysis was performed to estimate potential cost differences between the dosing schedules. We demonstrated that reducing the dose of bevacizumab would result in a significant cost saving, with no difference in overall survival outcomes, thereby potentially offering better value.

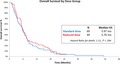

## 1 INTRODUCTION

Glioblastoma (GBM) has one of the highest mortality rates of any cancer. Following maximal surgical resection, radiation with concurrent and adjuvant temozolomide is standard.^1^ However, tumor progression inevitably occurs and second‐line treatment options are limited, with median survival ranging from 3 to 9 months.^2^, ^3^ Vascular proliferation is one of the pathological hallmarks of glioblastoma, which expresses high levels of the Vascular Endothelial Growth Factor Receptor (VEGFR).^4^, ^5^, ^6^ In progressive GBM the monoclonal antibody bevacizumab, which targets VEGFR, results in reduced tumor vascularity and vascular permeability.^7^ While there is some evidence patients appear to be living longer on average since its approval by the Federal Drug Administration (FDA) in the United States in 2009,^8^ bevacizumab has not yet shown an overall survival (OS) benefit in randomized phase III trials, and is not approved by the European Medicines Agency (EMA). An important benefit of bevacizumab in progressive GBM might be symptom control, since it can reduce cerebral edema with a resultant decrease in corticosteroid use.^9^, ^10^

10 mg/kg every 2 weeks (q2/52) bevacizumab was the standard dose used in early and subsequent trials.^9^, ^11^, ^12^, ^13^ However, it has since been suggested that a lower dose might offer similar benefits but with less toxicities and lower financial cost.^14^, ^15^, ^16^ Hence, the optimal bevacizumab dose is debated and has led to variable practice between Neuro‐Oncologists. In our institution, patients are randomly assigned on a rota system to one of three Neuro‐Oncologists, who use different doses of bevacizumab in their practices. In this retrospective study we examined whether bevacizumab dose had an impact on patient outcomes, analysed for potential predictive factors and performed a comparative cost analysis.

## 2 METHODS

This was a retrospective single‐institution study in the national neuro‐oncology tertiary referral centre in Ireland. Patients who received at least one dose of bevacizumab for progressive GBM between January 1, 2010 and January 1, 2017 were identified from the prospectively maintained patient database. All patients had received first‐line standard radiotherapy with concurrent and adjuvant temozolomide and were followed by a standard protocol prior to diagnosis of progression and commencing bevacizumab. Patients with de novo WHO Grade IV GBM only were included, while those with a history of WHO Grade II or III tumors who later progressed to GBM were excluded. As this study reflected everyday practice, patients were included irrespective of baseline performance status.

### 2.1 Study procedures

Data on patient demographics and tumor characteristics such as O^6^‐methylguanine DNA methyltransferase (MGMT) methylation status (≥9% vs <9%), Isocitrate Dehydrogenase 1 (IDH‐1) and ATRX mutation analysis were obtained from the institutions medical record database. Data on OS were obtained from the institution database and verified by review of medical records.

### 2.2 Dose‐levels

As this was a retrospective study, dose‐levels were pre‐defined by the different practices of the treating Neuro‐oncologists in our institution. No patient factors determined to which neuro‐oncologist the patients were randomly assigned, and therefore did not impact on the bevacizumab schedule received. For the purposes of this analysis, patients were grouped into standard‐dose (10 mg/kg q2/52 or 15 mg/kg q3/52) or reduced‐dose (5 mg/kg q2/52 or 7.5 mg/kg q3/52) bevacizumab. No patients changed dose schedule between the standard‐dose and reduced‐dose groups.

### 2.3 Endpoints

The primary endpoint was overall survival, measured from the date of first administered bevacizumab dose until death from any cause, among the standard‐dose and reduced‐dose bevacizumab cohorts. Secondary endpoints included age at diagnosis, time from diagnosis to bevacizumab start, gender, MGMT methylation, baseline PS and steroid use, and a comparative cost analysis. No attempt was made to retrospectively determine progression free survival (PFS) as pseudoprogression and radiation necrosis would have confounded accurate progression assessment.^17^, ^18^ Adverse events (AE's) such as hypertension, proteinuria, or thromboembolic events were not assessed due to the unreliable nature of retrospective documentation and the likelihood that patients had AE's treated elsewhere, eg hypertension with their primary care physician, given the tertiary care model in Ireland, thus making analyses inaccurate.

### 2.4 Exploratory analyses

Clinical status (stable/improved/worse vs previous visit), steroid use (yes/no & stable/increased/reduced dose vs previous visit), clinical benefit (any “stable” or “improved” clinical visit), steroid benefit (any reduction in steroid dose or stable steroid dose as the best response) and best clinical response (stable vs improved vs worse) were assessed by chart review and analysed as exploratory endpoints. Patients were subdivided into three groups to assess whether age at diagnosis impacted on OS; patients aged <50 years, patients aged between 50 and 65 years (ie 50 years ≤ GBM diagnosis ≤65 years), and patients aged >65 years at the time of their GBM diagnosis. Similarly, patients were subdivided into three groups (<12 months, 12‐18 months and >18 months after histological GBM diagnosis) to assess whether this impacted OS.

### 2.5 Statistical analysis

Standard descriptive statistics were employed to describe the entire study population, and both the standard‐ and reduced‐dose patient groups. To ensure comparability, Fisher or chi‐squared tests for categorical data and *t*‐test for continuous data were performed to assess for significant differences between the groups. A multivariate analysis for OS was planned if more than one univariate analysis reached statistical significance. Patients were censored at the time of death, the study endpoint (January 1, 2017) if still alive, or at their last confirmed hospital visit if lost to follow‐up and no definite date of death could be obtained. Survival curves were estimated by the Kaplan‐Meier method and compared using the log rank test (univariate analyses). OS rates at the predefined cut‐off points of 6‐, 12‐, and 24‐months were also compared with published data.

### 2.6 Cost analysis

Costs associated with bevacizumab administration at both standard‐dose and reduced‐dose were evaluated for the entire study period. Costs of bevacizumab were calculated based on the mean bevacizumab dose received by each patient across their treatment period. Bevacizumab was priced based on current costs in January 2019 in Ireland; €470.69 ($531.30) per 100mg vial, €1783.78 ($2013.48) per 400 mg vial. The combination of 400 mg and 100 mg vials which enabled minimal wastage was used to estimate costs in all cases. For example, for bevacizumab 700 mg, one 400 mg vial and three 100 mg vials were used, rather than two 400 mg vials.

## 3 RESULTS

### 3.1 Patient demographics

One hundred and eighteen (N = 118) patients received bevacizumab for progressive GBM during the study timeframe. Median age at diagnosis was 60 (Range: 18‐85) years with a male predominance (N = 77, 65.2%). Patients were predominantly Caucasian. The median time from histological GBM diagnosis to first bevacizumab treatment was 12.7 months (Range: 3.6‐54.3 months). 24 (20%) patients had a second surgery, 5 of which were for maximal safe resection after an initial biopsy‐only procedure, meaning 19 (16%) patients underwent re‐resection or debulking of their tumors prior to starting bevacizumab. MGMT Methylation status was assessable in 86/118 patients, 72.9% of the entire population. IDH‐1 mutation status was available in 90/118 patients (76.3%). There were no 1p/19q co‐deleted patients in the study population. ATRX mutation status was available in 47/118 patients (40%, Table 1).

**Table 1:** Baseline patient demographics for the entire population and comparing standard‐dose vs reduced‐dose groups

	Total population N (%)	Standard‐dose bevacizumab N (%)	Reduced‐dose bevacizumab N (%)	*P*‐value
Total Number of Patients	**118**	**69**	**49**	
Gender				
Male	77 (65)	45 (65)	32 (65)	.99
Female	41 (35)	24 (35)	17 (35)	
Further debulking surgeries prior to bevacizumab start				
1 Re‐resection	24 (20)	19 (27.5)	5 (10)	
2 Re‐resections	1 (1)	0	1 (2)	
MGMT methylation Status	**Known (86/118)**	**Known (50/69)**	**Known (36/49)**	
Methylated	37 (43)	20 (40)	17 (47)	.50
Unmethylated	49 (57)	30 (60)	19 (53)	
IDH‐1 status	**Known (90/118)**	**Known (53/69)**	**Known (37/49)**	
Mutated	6 (7)	4 (7.5)	2 (5.1)	.69
Wild‐type	84 (93)	49 (92.5)	35 (94.9)	
1p/19q Co‐deletion				
Co‐deleted	0	0	0	N/A
Time from diagnosis to bevacizumab start (mo)	**N = 118**	**N = 69**	**N = 49**	
<12	60 (51)	36 (52)	24 (49)	.665
12‐18	28 (24)	16 (23)	12 (24)	
>18	30 (25)	17 (25)	13 (27)	
Age at GBM diagnosis (y)				
<50	31 (26)	14 (20)	17 (35)	.173
50‐65	57 (48)	38 (55)	19 (39)	
>65	30 (25)	17 (25)	13 (26)	
Median (y, Range)	59.4 (16‐85)	58.9 (22‐82)	60.9 (16‐85)	.95
ECOG PS at baseline	**N = 75**	**N**	**N**	
Total recorded	15	10	5	
ECOG PS 0	0	0	0	
ECOG PS 1	8	6	2	
ECOG PS 2	5	3	2	
ECOG PS 3	2	1	1	

In total, 1,127 bevacizumab doses were administered over the study period (Median: 7, Range: 1‐44 per patient). Clinical data from chart review were available in 75/118 (64%) patients. Of these, 53/75 (71%) patients had available data on baseline steroid use with 34/53 (64%) taking steroids at baseline. Evaluable information on steroid dose was available from 607 treatment visits and on clinical status was available from 716 treatment visits (Table S1).

69 (59%) patients received standard‐dose bevacizumab and 49 (41%) patients received reduced‐dose bevacizumab. Both groups were similar with regards to age range, gender, time from diagnosis, MGMT methylation status %, time from diagnosis to bevacizumab start, IDH mutation status and ATRX alteration status (Table 1 and Tables S1,S2). 568 standard‐dose bevacizumab cycles were administered (Median: 6, Range: 1‐36) while 549 reduced‐dose bevacizumab cycles were administered (Median: 8, Range: 1‐44; Table S1).

### 3.2 Overall survival, entire population

Median OS (mOS) from time of first bevacizumab administration was 5.8 months (Range: 0.5‐41) for the entire population with OS rates of 47.5%, 20.25% & 4.3% at 6‐, 12‐ & 24‐months. Male patients (N = 77) had a numerical but not statistical OS advantage over female patients (N = 41), (mOS: 6.4 vs 5.2 months, HR: 0.80, *P* = .28). Median OS from time of initial histological diagnosis to death was 19.4 months (Range: 6‐59.9).

### 3.3 Overall survival by dose group

**Figure 1:**
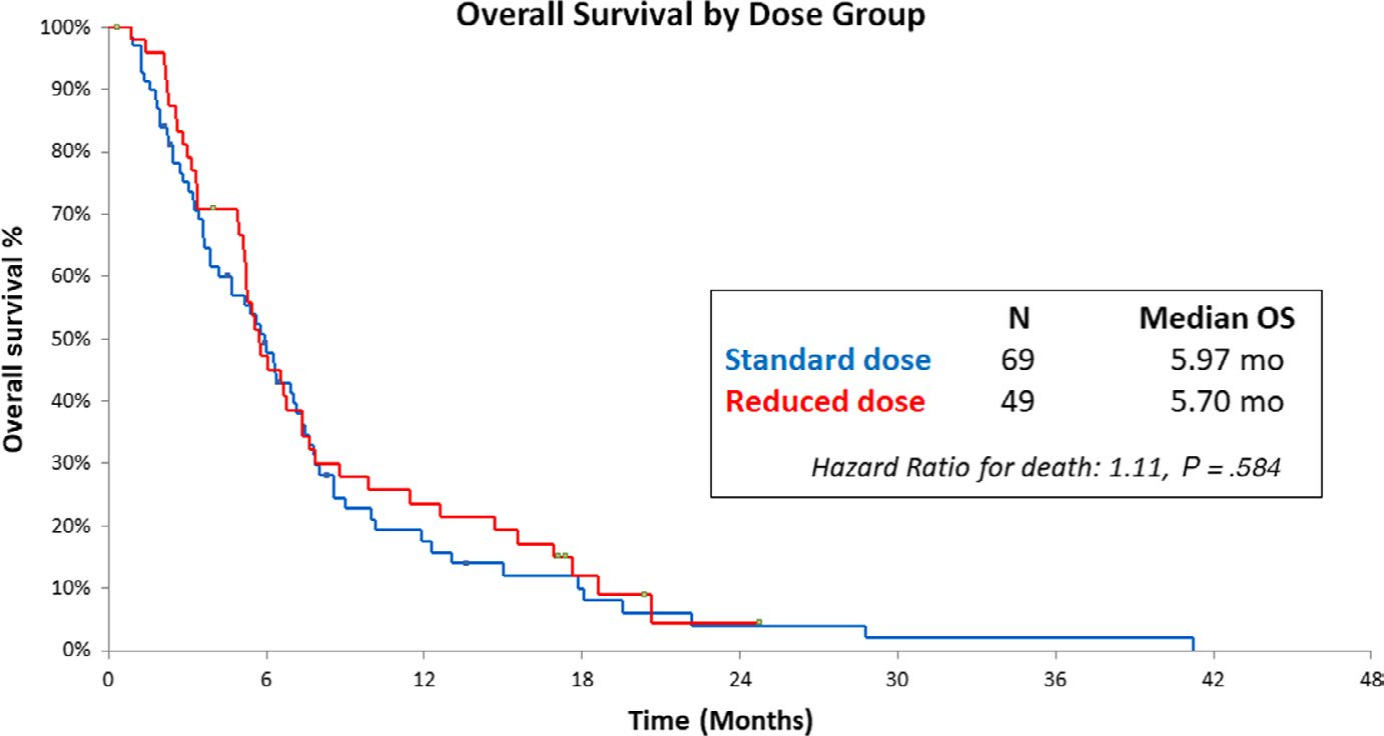
Kaplan‐Meier estimates of overall survival, according to dose group

Median OS was similar in patients who received standard‐dose (5.97 months) and reduced‐dose (5.7 months) bevacizumab (Δ 0.27 months, HR: 1.11, *P*‐value: .584, Figure 1). Similar OS was seen at 6‐months (47.7% vs 47.2%), 12‐months (23.6% vs 17.5%) and 18‐months (12% vs 10%) in both bevacizumab cohorts. Univariate analysis showed no impact of patient and tumor characteristics on median OS (Table S2).

### 3.4 Impact of tumor characteristics on overall survival

**Figure 2:**
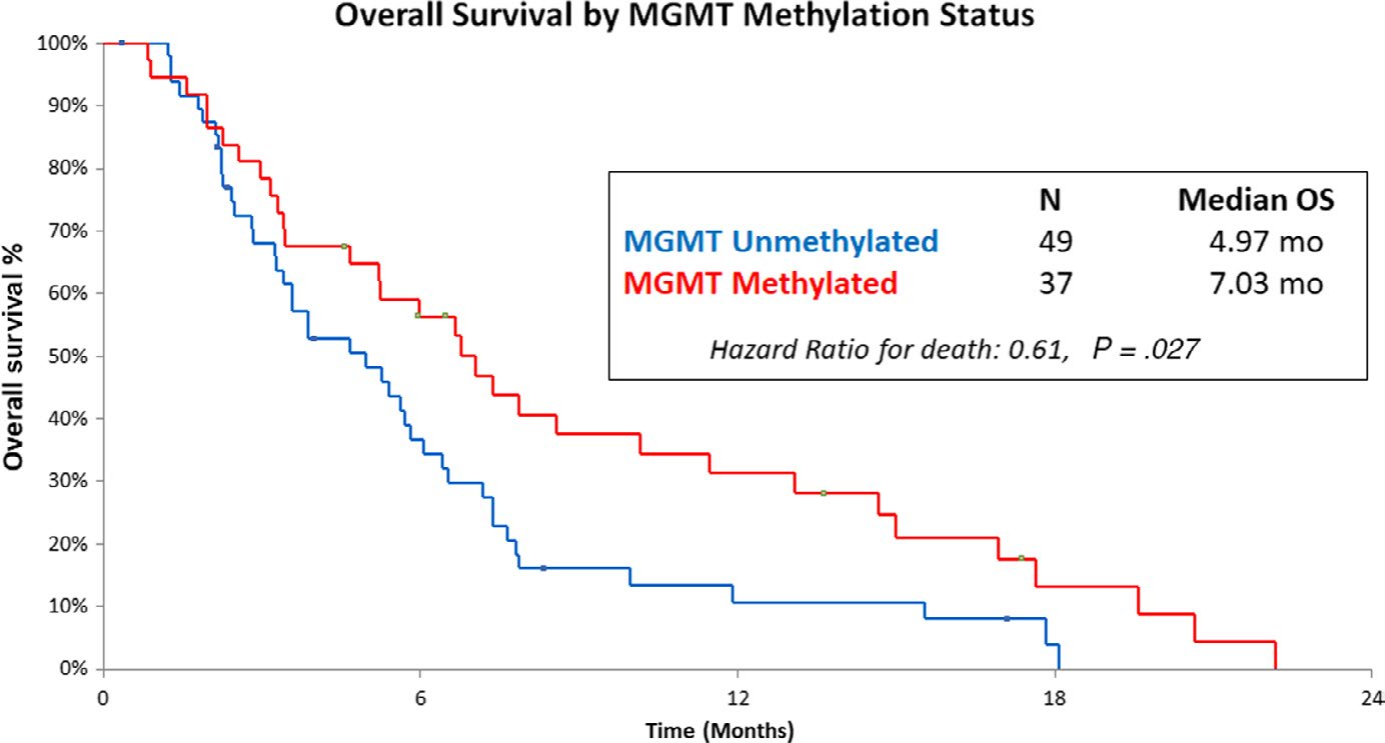
Kaplan‐Meier estimates of overall survival, according to MGMT promoter methylation status

37/86 (43%) assessable patients for MGMT methylation status had MGMT methylated tumors (mOS: 7.03 months) which was statistically significant when compared to unmethylated tumors (49/86, mOS: 4.97 months), HR: 0.61, *P*‐value: .027 (Figure 2). The majority of patients with available IDH‐1 mutation status (N = 90) were IDH‐1 wild‐type (N = 84, 94.9%; mOS: 5.47 months) while 6 patients (5.1%) had IDH‐1 mutated tumors, (mOS: 5.97 months), HR 1.38, *P*‐value .436. 6 patients (12.8%) had ATRX mutated tumors while 41 were ATRX wild‐type of 47 assessable tumors (mOS: 5.0 vs 6.0 months, HR 0.65, *P*‐value .357; Table S2).

### 3.5 Clinical status and steroid use by dose group

Higher proportions of “stable” (82.3% vs 74.5%) and lower rates of “worse” (11.9% vs 18.1%) clinical status were seen in the reduced dose bevacizumab group vs the standard dose bevacizumab group, respectively, while the number of visits where the patients clinical status “improved” was low in both groups (7.4% vs 5.8%, Figure S1). Steroids were “decreased” more commonly in the standard vs reduced‐dose cohorts (16.8% vs 11.2%), but also “increased” more commonly (14.4% vs 10.4%, Figure S2).

Best clinical response assessment identified 41.4% of patients as having an “improved” clinical status between any two treatment visits while on bevacizumab. 48.6% of patients had “stable” best clinical response while 10% of patients were “worse” as their best clinical response (Figure S3). Numerical but not statistical differences were seen between the standard and reduced‐dose cohorts (Table S3).

Clinical Benefit was seen in 91% of the entire population (63/69 evaluable patients). Rates were slightly higher in the reduced‐dose (31/32, 97%) vs the standard‐dose cohort (32/37, 86%), *P* = .205. Steroid Benefit was seen in 95% (59/62) of the entire evaluable population. 29/29 patients in the reduced dose bevacizumab group had a steroid benefit, while 30/33 (91%) of the standard dose bevacizumab group achieved a steroid benefit (*P* = .241, Table S3).

### 3.6 Age at diagnosis and time from diagnosis to bevacizumab start as predictive markers

31 patients were <50 at the time of diagnosis (mOS: 5.80 months), 57 patients were aged between 50‐65 years (mOS: 5.80 months) and 30 patients were aged >65 (mOS: 6.07 months), common *P*‐value: 0.173 (Figure S4). 59 patients (50%) commenced bevacizumab within 12 months of histological GBM diagnosis (mOS: 5.3 months), 29 patients commenced bevacizumab between 12‐18 months (mOS: 6.3 months), while 30 patients commenced bevacizumab at >18 months from histological GBM diagnosis (mOS: 6.8 months), common *P*‐value: .665 (Figure S5).

### 3.7 Cost analysis

The total cost of bevacizumab administration across all patients for the duration of the study was €3,524,106.18 ($3,986,821.32). Among the 49 patients who received reduced‐dose bevacizumab, total cost savings over the entire study period was €1,272,629.79 ($1,439,726.08). If reduced‐dose bevacizumab were applied to all 118 patients in the study, a cost reduction of €2,398,367.99 ($2,713,273.71) would be observed (Table S4).

## 4 DISCUSSION

In this retrospective study, OS was statistically similar (5.97 vs 5.7 months; HR: 1.11, *P*‐value: .584) with no clinically significant difference (Δ 0.27 months) among patients with progressive GBM who received standard‐dose (N = 69) or reduced‐dose (N = 49) bevacizumab. This homogenous WHO Grade IV patient population appear well balanced across all patient demographics assessed between the two dose cohorts, as demonstrated by univariate analysis (Tables 1 and Tables S1,S2). This suggests that reducing the bevacizumab dose‐schedule has minimal or no impact on efficacy and that a reduced dose might be a preferable, cheaper option. Although this was not a prospective phase III trial, bevacizumab dose schedule was randomly determined based on the on‐call system between three Neuro‐Oncologists. We believe this limits the impact of selection bias on our conclusions.

10 mg/kg q2/52 was the standard‐dose used in early and subsequent trials, where median OS results varied from 3.9‐10 months (31‐42 weeks), however bevacizumab was often administered in combination with Irinotecan or Lomustine.^9^, ^11^, ^12^, ^13^, ^19^ Increasing evidence however suggests 10 mg/kg q2/52 may not be the optimum dose‐schedule. Traditional dose‐setting tools such as dose‐limiting toxicity (DLT) and maximum tolerated dose (MTD) are inadequate for monoclonal antibodies such as bevacizumab that target cell surface antigens, exhibit nonlinear pharmacokinetics due to receptor‐mediated clearance and where activity can change due to disease severity or receptor loss following repeated dosing.^20^, ^21^, ^22^

Studies investigating a lower dose bevacizumab regimen hypothesized this would offer similar benefits and lower toxicities. Lorgis et al 14 concluded that a lower dose (<5 mg kg^−1^ wk^−1^) had improved OS over standard‐dose bevacizumab (≥5 mg kg^−1^ wk^−1^) among 110 consecutive patients, but in a heterogenous population of progressive WHO Grade III or IV glioma patients (mOS: 16 vs 6 months, *P* = .0002). The majority of patients included also received concurrent irinotecan (85.5% vs 96%, test vs validation cohorts) and differences between the study arms were seen; WHO Grade IV patients (72% vs 89%), Karnofsky Class ≥70% (55% vs 70%), previous temozolomide regimen (68% vs 85%). In contrast, Blumenthal et al^15^ found no OS difference in 162 patients treated with bevacizumab 5 mg/kg q2/52 (N = 87, mOS 7.1 mo) vs 10 mg/kg q 2/52 (N = 75, mOS 7.8 mo), however 65.5% of the 5 mg/kg cohort received concurrent chemotherapy compared to 20% of the 10 mg/kg cohort. Ajlan et al^16^ noted lower toxicity rates with low‐dose bevacizumab monotherapy (<3 mg kg^−1^ wk^−1^) vs high dose bevacizumab (>3 mg kg^−1^ wk^−1^) and a trend toward improved OS rates but that was not statistically significant in a cohort of 80 patients. Interestingly, as shown by Ajlan et al,^16^ we noted that a higher number of treatments were administered to patients in the reduced‐dose group (median 8 vs 6), which may reflect better tolerability as they had suggested, or a decreased propensity to “normalize” tumor vasculature, as has been suggested elsewhere.^14^, ^23^

Given the cost of bevacizumab, these results have important implications for value in cancer care. Our cost analysis revealed that decreasing from standard (10 mg/kg) to reduced‐dose (5 mg/kg) bevacizumab for 49 patients resulted in an estimated €1.3M saving. If all patients (N = 118) were treated with reduced‐dose bevacizumab schedule, a €2.4M cost saving would be observed (Table S4). While there are increased drug administration costs associated with an increased number of treatment cycles, this cost saving would undoubtedly represent better value.

Additionally, we identified a statistically significant OS benefit (HR 0.61, *P* = .027) for patients whose tumors had methylated MGMT promoters (Figure 2). To the best of our knowledge this has not previously been reported. This likely reflects favorable tumor biology and suggests that MGMT remains an important prognostic, but less important predictive, biomarker.^24^

This retrospective study in a Western European predominantly Caucasian population was carried out in a national neuro‐oncology centre with expertise in treating primary CNS tumors. Our results, therefore, may not be generalizable to other centres and this limitation should be taken into account when analysing our findings. Furthermore, due to the time span (2010‐2017) and retrospective nature of the study, some patient records were not evaluable for steroid and clinical status analyses as they lacked reliable documentation or did not include adequate serial data and were therefore excluded. As a result, our steroid data and clinical status data sets and results reflect only a subset (64%) of the total population. Formal quality of life (QOL) indices and questionnaires were not applied routinely or consistently across our cohorts either and therefore were considered unreliable for valid retrospective analyses. These elements should be applied in future prospective studies in this field.

In conclusion, we feel two important points have been proven in this study. Firstly, reduced‐dose bevacizumab has a similar OS to standard‐dose bevacizumab monotherapy and is associated with a substantial cost saving. Secondly, MGMT methylation appears to convey a survival benefit even in the setting of bevacizumab treatment in progressive GBM. This study should help guide current clinical practice, future clinical studies and help improve value in cancer care.

## CONFLICT OF INTEREST

JPG and FK have received Honoraria for unrelated work from Roche Pharmaceuticals. EH has received travel support from Roche Pharmaceuticals. PGM has received travel support and honoraria from Roche Pharmaceuticals. NMK, EM, AA, JH, AEL, POH, SMN, BTH, OSB, & LG report no relevant disclosures or conflicts of interest.

## AUTHOR CONTRIBUTIONS

JPG and PGM were involved in all aspects of the study concept & design, data acquisition & analysis, drafting of the manuscript and manuscript submission. FK contributed to drafting the manuscript and performed the cost analysis. NMK, EM, EH, AA, JH, AEL, & POH contributed to the acquisition of data. SMN, BTH, OSB, & LG contributed to the study concept & design and were the primary care providers for patients included in the study.

## ETHICS REVIEW

Prior to initiation the study was reviewed at the Oncology Research meeting. Since this was a retrospective study it was determined that ethics/IRB approval was not required as per standard institutional practice.
