# Supplementary figures and images for: Similar overall survival with reduced vs. standard dose bevacizumab monotherapy in progressive glioblastoma
Source: Cancer Med. 2019 Nov 22;9(2):469–75. doi: 10.1002/cam4.2616 (PMC6970030; doi:10.1002/cam4.2616)

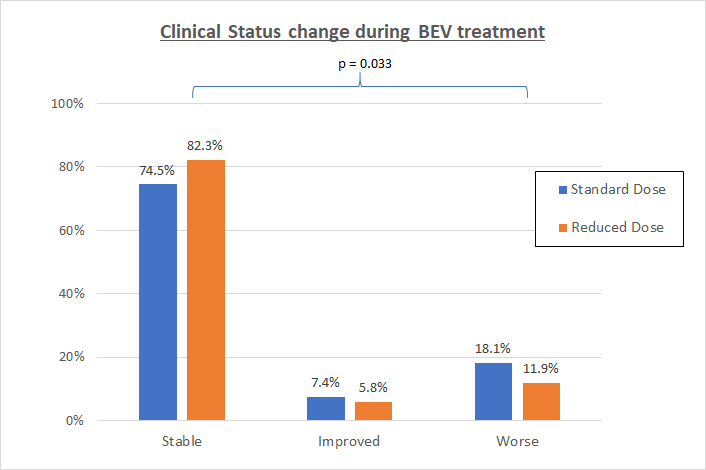

Supplement: Supplementary file 1 [file CAM4-9-469-s001.tif]

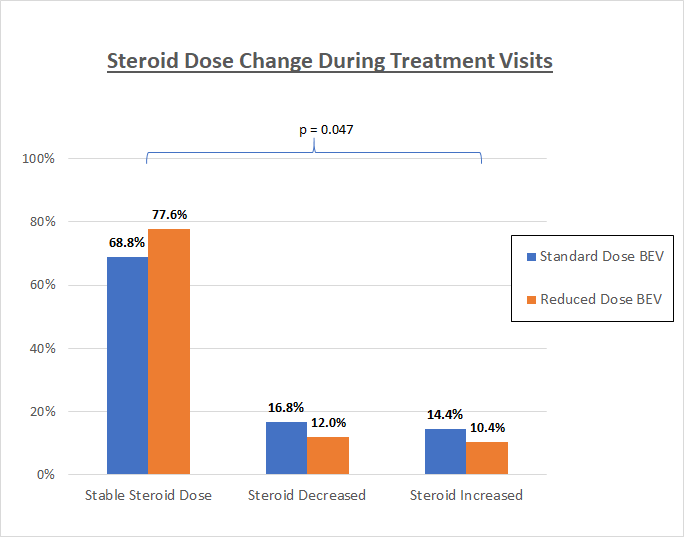

Supplement: Supplementary file 2 [file CAM4-9-469-s002.tif]

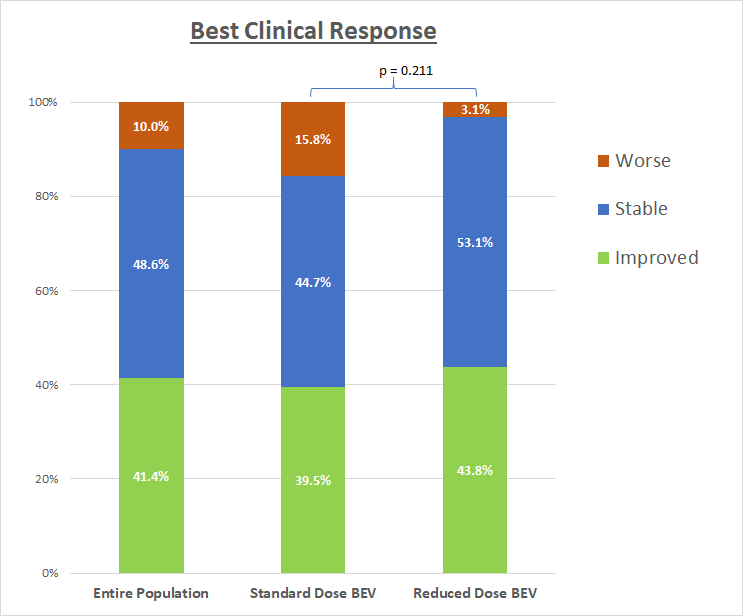

Supplement: Supplementary file 3 [file CAM4-9-469-s003.tif]

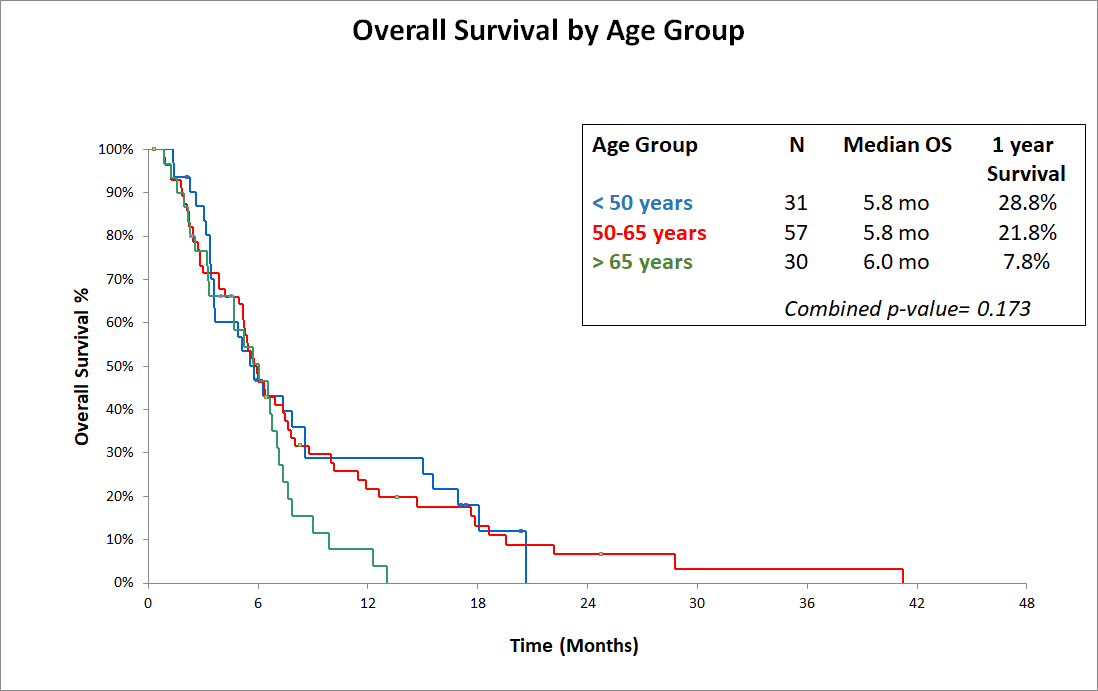

Supplement: Supplementary file 4 [file CAM4-9-469-s004.tif]

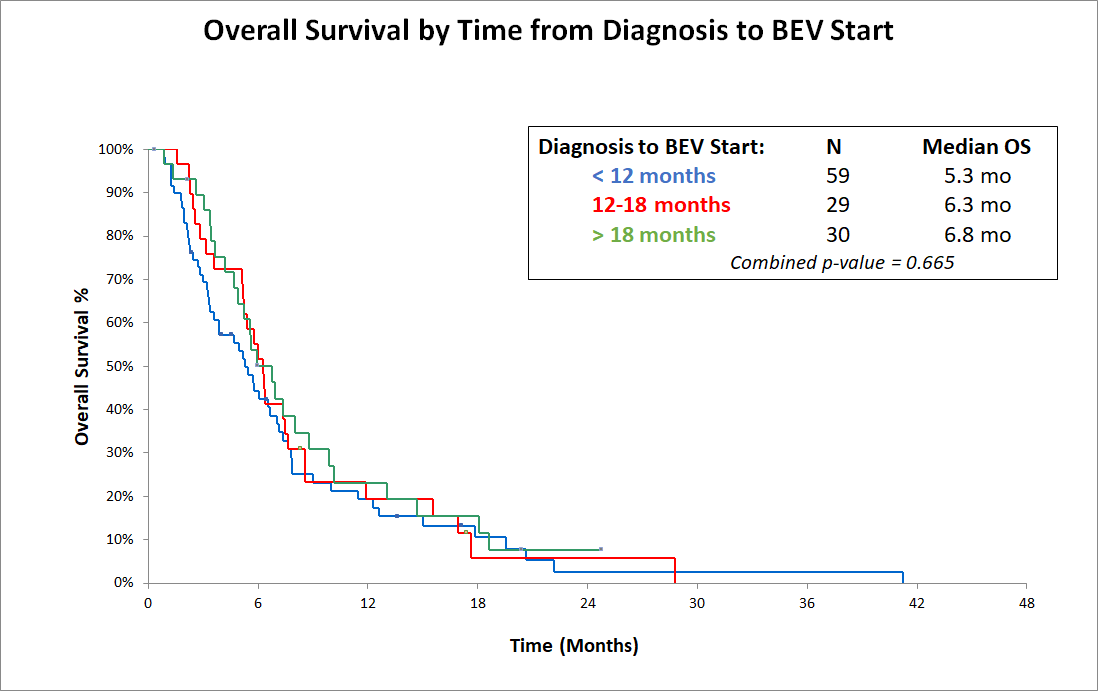

Supplement: Supplementary file 5 [file CAM4-9-469-s005.tif]
