# Supplementary material for: Similar overall survival with reduced vs. standard dose bevacizumab monotherapy in progressive glioblastoma
Source: Cancer Med. 2019 Nov 22;9(2):469–75. doi: 10.1002/cam4.2616 (PMC6970030; doi:10.1002/cam4.2616)
Supplement: Supplementary file 6 [file CAM4-9-469-s006.docx]

SUPPLEMENTARY TABLE 1. Bevacizumab Treatment demographics by total population and by Standard- and Reduced-dose Bevacizumab groups.

|  | Total Population | Standard-DOSE Bevacizumab | REduced-Dose BEVacizumab |
| --- | --- | --- | --- |
|  | ***N* (%)** | ***N* (%)** | ***N* (%)** |
| Total Number of Patients | **118** | **69** | **49** |
| Number of Treatment Visits |  |  |  |
| Total Visits | 1127 | 568 | 549 |
| Median Per Patient | 6 | 6 | 8 |
| Mean Per Patient | 9.55 | 8.23 | 11.4 |
| Evaluable Patients for Clinical Status & Steroids, *N* (%) | **75 (63.6)** | **41 (59)** | **34 (69)** |
| Clinical Change During Treatment Versus Previous Visit |  |  |  |
| Total Evaluable Patient Treatment Visits, *N* (%) | 716 (63.5) | 337 (59.3) | 379 (69) |
|  |  |  |  |
| Improved | 47 (6.6) | 25 (7.4) | 22 (5.8) |
| Stable | 563 (78.6) | 251 (74.5) | 312 (82.3) |
| Worse | 106 (14.8) | 61 (18.1) | 45 (11.9) |
| Steroid Use at Baseline | **Evaluable *N*=75** | ***N* (% Total)** | ***N* (% Total)** |
| Yes | 34 (64) | 20 (62.5) | 14 (66.7) |
| No | 19 (36) | 12 (37.5) | 7 (33.3) |
| Not recorded | 22 | 9 | 13 |
| Steroid Dose Change During Treatment Versus Previous Visit |  |  |  |
| Total Evaluable Patient Treatment Visits, *N* | 607 | 298 | 309 |
|  |  |  |  |
| Steroid Reduced | 87 (14.3) | 50 (16.8) | 37 (12.0) |
| Stable Dose | 445 (73.3) | 205 (68.8) | 240 (77.6) |
| Steroid Increased | 75 (12.4) | 43 (14.4) | 32 (10.4) |
| Treatment beyond progression with bevacizumab |  |  |  |
| Number of patients | 6 (5.1) | 3 (4.3) | 3 (6.1) |
| Temozolomide + Bevacizumab | 4 (3.4) | 2 (2.9) | 2 (4.1) |
| Lomustine | 2 (1.7 | 1 (1.4) | 1 (2.0) |
