# Supplementary material for: Similar overall survival with reduced vs. standard dose bevacizumab monotherapy in progressive glioblastoma
Source: Cancer Med. 2019 Nov 22;9(2):469–75. doi: 10.1002/cam4.2616 (PMC6970030; doi:10.1002/cam4.2616)
Supplement: Supplementary file 7 [file CAM4-9-469-s007.docx]

| **Patient/Tumour Characteristic** | **Standard Dose**  **Bevacizumab** | | **Reduced Dose**  **Bevacizumab** | | **HR, *p*-value** | **Total Population** | | **HR, *p*-value** |
| --- | --- | --- | --- | --- | --- | --- | --- | --- |
|  | ***N*** | **mOS** | ***N*** | **mOS** |  | ***N*** | **mOS** |  |
| **Gender**  - Male  - Female | 45  24 | 6.4  5.2 | 32  17 | 6.5  5.2 | 1.09, 0.719  1.11, 0.748 | 77  41 | 6.4  5.2 | 0.8, 0.280 |
| **Age Group (at Dx)**  - < 50 years  - 50-65 years  - > 65 years | 14  38  17 | 6.3  5.8  4.7 | 17  19  13 | 5.5  5.5  6.1 | 1.35, 0.424  1.08, 0.780  0.71, 0.345 | 31  57  30 | 5.8  5.8  6.0 | NA, 0.151 |
| **Time from Dx to Bevacizumab start**  - < 12 months  - 12-18 months  - > 18 months | 36  16  17 | 4.7  6.3  6.0 | 23  13  13 | 5.7  5.2  6.8 | 0.91, 0.727  0.97, 0.925  1.83, 0.119 | 59  29  30 | 5.3  6.3  6.8 | NA, 0.665 |
| **Steroid use at baseline**  - Yes  - No | 20  12 | 6.0  8.6 | 14  7 | 5.3  8.8 | 0.93, 0.842  0.87, 0.767 | 34  19 | 5.7  8.8 | 1.31, 0.363 |
| **MGMT**  - Methylated  - Unmethylated | 20  30 | 7.0  3.9 | 17  19 | 6.8  5.3 | 0.98, 0.945  0.97, 0.920 | 37  49 | 7.0  5.0 | **0.61, 0.027** |
| **IDH1**  - Mutated  - Wildtype | 4  49 | 15.0  4.7 | 2  35 | 2.3  5.8 | 0.56, 0.493  1.50, 0.081 | 6  84 | 15.0  5.5 | 0.59, 0.247 |

**SUPPLEMENTARY TABLE 2.** Impact of patient and tumour characteristics on OS – univariate analysis showing median OS (months) across subgroups, comparison between standard and reduced-dose Bevacizumab on OS and for the entire population.
