# Supplementary material for: Similar overall survival with reduced vs. standard dose bevacizumab monotherapy in progressive glioblastoma
Source: Cancer Med. 2019 Nov 22;9(2):469–75. doi: 10.1002/cam4.2616 (PMC6970030; doi:10.1002/cam4.2616)
Supplement: Supplementary file 8 [file CAM4-9-469-s008.docx]

**SUPPLEMENTARY TABLE 3.** Best recorded response during treatment with Bevacizumab.

|  | **Entire Population** | | **Reduced-Dose Bevacizumab** | | **Standard-Dose Bevacizumab** | |  |
| --- | --- | --- | --- | --- | --- | --- | --- |
|  | *N* | % | *N* | % | *N* | % | ***p-value^a^*** |
| **Best Clinical Response:** |  | |  | |  | |  |
| Patients with evaluable data, % of entire population | 69 | 58 | 32 | 65 | 37 | 54 |  |
|  |  |  |  |  |  |  |  |
| Improved | 29 | 42 | 14 | 44 | 15 | 40 | 0.308 |
| Stable | 34 | 49 | 17 | 53 | 17 | 46 |  |
| Worse | 6 | 9 | 1 | 3 | 5 | 14 |  |
|  |  |  |  |  |  |  |  |
| **Clinical Benefit^b^ %** | 63/69 | 91 | 31/32 | 97 | 32/37 | 86 | 0.205 |
|  |  |  |  |  |  |  |  |
| **Best Steroid Response:** |  |  |  |  |  |  |  |
| Patients with evaluable data, % of entire population | 62 | 53 | 29 | 59 | 33 | 48 |  |
|  |  |  |  |  |  |  |  |
| Dose Decreased | 38 | 61 | 16 | 55 | 22 | 67 | 0.145^d^ |
| Dose Stable | 21 | 34 | 13 | 45 | 8 | 24 |  |
| Dose Increased | 3 | 5 | 0 | 0 | 3 | 9 |  |
|  |  |  |  |  |  |  |  |
| **Steroid Benefit^c^ %** | 59/62 | 95 | 29/29 | 100 | 30/33 | 91 | 0.241 |
|  |  |  |  |  |  |  |  |

^a^ p-value for comparison of Standard-dose vs. Reduced-dose Bevacizumab displayed.

^b^ Clinical Benefit defined as clinically improved or stable as a best clinical response.

^c^ Steroid Benefit defined as a decrease in steroid dose or maintenance on the same dose of steroids between clinical visits.

^d^ *p*-value for Dose decreased vs. Dose Increased displayed, as no patients in the Reduced-Dose Bevacizumab group had a steroid dose increase as the best response.
