# Supplementary material for: Similar overall survival with reduced vs. standard dose bevacizumab monotherapy in progressive glioblastoma
Source: Cancer Med. 2019 Nov 22;9(2):469–75. doi: 10.1002/cam4.2616 (PMC6970030; doi:10.1002/cam4.2616)
Supplement: Supplementary file 9 [file CAM4-9-469-s009.docx]

|  | Standard-DOSE BEVacizumab | REduced-Dose BEVacizumab | Total Population |
| --- | --- | --- | --- |
|  | ***N* (%)** | ***N* (%)** | ***N* (%)** |
| Total Number of Patients | **69 (58.5)** | **49 (41.5)** | **118 (100)** |
| Bevacizumab Costs Over Entire Study Period (84 months) |  |  |  |
| Total Cost (€) | 2,251,476.39 | 1,272,629.79 | **3,524,106.18** |
| Average Cost Per Patient | 32,630.09 | 25,972.04 | 29,865.31 |
| Average Number of Cycles Received per Patient | 8.23 | 11.4 | 9.55 |
| Actual Cost Reduction over Entire Study Period for Reduced-Dose Patients (84 months) |  |  |  |
| Total Cost Reduction(€) | - | 1,272,629.79 | - |
| Average Cost Reduction per patient (€) | **-** | 25,972.04 | **-** |
| Potential Cost Reduction over Entire Study Period if All Patients had received Reduced-Dose instead of Standard-Dose (84 months) |  |  |  |
| Total Cost Reduction (€) | 1,125,738.2 | 1,272,629.79 | **2,398,367.99** |
| Average Cost Reduction per patient (€) | 16,315.05 | 25,972.04 | **20,325.15** |

SUPPLEMENTARY TABLE 4: Cost analysis of Bevacizumab administration, with actual and potential cost reductions over entire study period, comparing standard-dose vs. reduced-dose groups.
